# The Association Between Cephalosporin and Hypoprothrombinemia: A Systematic Review and Meta-Analysis

**DOI:** 10.3390/ijerph16203937

**Published:** 2019-10-16

**Authors:** Gi Hyue Park, Seungyeon Kim, Min Soo Kim, Yun Mi Yu, Gun Hee Kim, Jeong Sang Lee, Euni Lee

**Affiliations:** 1 College of Pharmacy & Research Institute of Pharmaceutical Sciences, Seoul National University, Seoul 08826, Korea; gihyuepark@snu.ac.kr (G.H.P.); rlatmdus92@gmail.com (S.K.); min1247419@gmail.com (M.S.K.); 54379@snubh.org (G.H.K.); 2 Department of Pharmacy and Yonsei Institute of Pharmaceutical Sciences, College of Pharmacy, Yonsei University, Incheon 21983, Korea; yunmiyu@yonsei.ac.kr; 3 Department of Pharmaceutical Medicine and Regulatory Sciences, Colleges of Medicine and Pharmacy, Yonsei University, Incheon 21983, Korea; 4 Department of Thoracic & Cardiovascular Surgery, SNU-SMG Boramae Hospital, Seoul 07061, Korea; 5 Department of Thoracic & Cardiovascular Surgery, College of Medicine, Seoul National University, Seoul 07061, Korea

**Keywords:** cephalosporins, hypoprothrombinemia, prothrombin time, bleeding

## Abstract

Cephalosporins that contain the N-methylthiotetrazole side chain (NMTT-cephalosporin) have been reported to be associated with coagulation-related adverse events; however, a comprehensive evaluation regarding the association is lacking. A systematic review and meta-analysis were conducted to assess the safety profile of NMTT-cephalosporins with respect to hypoprothrombinemia and bleeding. The MEDLINE, Embase, Cochrane, and RISS databases were systematically searched for clinical studies up to October 2018. The association between NMTT-cephalosporins and hypoprothrombinemia was estimated using an odds ratio (OR) with a 95% confidence interval (CI). A total of 15 studies on cefamandole, cefoperazone, cefotetan, cefmetazole, and moxalactam were identified and included in the meta-analysis. Hypoprothrombinemia (OR 1.676, 95% CI 1.275–2.203) and prothrombin time (PT) prolongation (OR 2.050, 95% CI 1.398–3.005) were significantly associated with NMTT-cephalosporins, whereas bleeding was not (OR 1.359, 95% CI 0.920–2.009). Subgroup analyses revealed that cefoperazone (OR 2.506, 95% CI 1.293–4.860), cefamandole (OR 3.247, 95% CI 1.083–9.733), and moxalactam (OR 3.367, 95% CI 1.725–6.572) were significantly associated with hypoprothrombinemia. An Antimicrobial Stewardship Program led by a multidisciplinary team could play a critical role in monitoring cephalosporin-related hypoprothrombinemia or PT prolongation in patients with underlying clinical conditions at risk for bleeding. The multidisciplinary team could also assist in communicating the potential safety concerns regarding NMTT-cephalosporin use with healthcare professionals to decrease the risk of adverse events.

## 1. Introduction

Cephalosporins are one of the most commonly used antibiotics in clinical practice due to their relatively safe and broad-spectrum activity against both Gram-positive and -negative bacteria. Moreover, the dosage of cephalosporins can easily be converted between intravenous and oral administration [1]. Due to these advantages, cephalosporins are the most frequently prescribed antibiotics in South Korea [2]. However, a majority of these drugs are not approved in other countries [3].

Cephalosporins are classified into four major generations, depending on their antimicrobial spectrum coverage [4]. Cephalosporins containing an *N*-methylthiotetrazole (NMTT) side chain are mainly the second-generation (cefamandole, cefbuperazone, cefmetazole, cefminox, cefotetan) and third-generation cephalosporins (cefmenoxime, cefoperazone, moxalactam). Of these eight NMTT-cephalosporins, six cephalosporins including cefamandole, cefmenoxime, cefmetazole, cefoperazone, moxalactam, and cefotetan had been approved by the United States (US) Food and Drug Administration (FDA), but only cefotetan is currently marketed; whilst six cephalosporins (cefotetan, cefamandole, cefmetazole, cefmenoxime, cefoperazone, and moxlactam [latamoxef]) have been approved and are currently marketed in South Korea. 

NMTT-cephalosporins are known to cause hemostatic abnormalities, such as bleeding, prothrombin time (PT) prolongation, and hypoprothrombinemia, due to the chemical structure of the NMTT, which interferes with vitamin K metabolism [5]. While the association between NMTT-cephalosporins and hypoprothrombinemia has been reported since the 1980s, evidence demonstrating the direct causal link has been conflicting. Early studies in the 1980s proposed that NMTT-cephalosporins directly induced hypoprothrombinemia [6], but some comparative studies from the same period reported that the direct connection between NMTT-cephalosporins and an increased risk for hematologic adverse effects was controversial [7]. Currently, the majority of the literature supporting the association between NMTT-cephalosporins and hypoprothrombinemia was based on observational studies and case reports. In particular, studies comparing the risk for hypoprothrombinemia between NMTT- and non-NMTT-cephalosporins are even scarcer.

Due to the lack of evidence regarding the clinical significance of hemorrhagic adverse events associated with one of the most prevalently used antibiotics and the paucity of comprehensive evaluation, we performed a systematic review and meta-analysis to determine the safety profile of NMTT-cephalosporins in terms of the risk for hypoprothrombinemia, PT prolongation, and bleeding as adverse events.

## 2. Materials and Methods

### 2.1. Search Strategy

We conducted a comprehensive systematic review of the literature to identify articles that compared the incidence of hypoprothrombinemia, abnormalities of coagulation tests, and bleeding between NMTT-cephalosporins and non-NMTT-cephalosporins published up to October 2018, using the following databases: MEDLINE, Embase, Cochrane Central Register of Controlled Trials, and Research Information Sharing Service (RISS). Case reports, letters, and conference abstracts were excluded. No language restriction was applied. The references cited in selected articles were also reviewed to include any relevant publications. The following search terms were used: (i) ‘cephalosporin’ OR the generic names of individual NMTT-cephalosporins, (ii) ‘hypoprothrombinemia’, and (iii) ‘bleeding’ OR ‘hemorrhage’ (Table S1). A designated researcher (GHP) identified the articles according to the search strategy described above. 

### 2.2. Eligibility Criteria and Study Selection

Studies meeting the following selection criteria were included in this systematic review and meta-analysis: studies that included participants who were aged ≥18 years and underwent single NMTT-cephalosporin therapy and studies that contained terms indicating hypoprothrombinemia, or those that reported laboratory or clinical indicators of hypoprothrombinemia, such as increased PT and adverse drug reactions (ADRs) of bleeding. The following studies were excluded: in vitro or in vivo studies, pediatric studies, studies with combination therapy, studies reporting not directly related outcomes, inappropriate comparison, such as absence or unrelated comparison groups, and studies with no access to their full-text articles. 

One researcher (GHP) searched the related articles according to the search strategy, and a second researcher (SK) confirmed the search process. After removing duplicates, two researchers (GHP and SK) independently selected studies by reviewing the titles, abstracts, and full texts, based on the aforementioned eligibility criteria. Any disagreements between the reviewers were resolved by consensus involving the participation of a third investigator (MSK).

### 2.3. Data Extraction and Quality Assessment

The following information was extracted using a standardized form: country, year of publication, study design, population characteristics, number of total population, generic names of NMTT-cephalosporins as intervention and control, dosage, and study outcome. Most of the studies we came across identified patients using medical records. If available, we recorded the odds ratios (ORs), as well as the number or proportion of patients who experienced hypoprothrombinemia, PT prolongation, or bleeding events, which can be converted into ORs.

For quality assessment, the Cochrane Collaboration Risk of Bias (RoB) tool (Table S2) [8] and modified Newcastle–Ottawa Scale (NOS) (Table S3) [9] were used for randomized controlled trials (RCTs) and observational studies, respectively. The RoB tool, comprising seven domains, was used; each domain was assessed as *high*, *low*, or *unclear* of bias: (1) random sequence generation, (2) allocation concealment, (3) blinding of participants and personnel, (4) blinding of outcome assessment, (5) incomplete outcome data addressed, (6) free of selective reporting, and (7) free of other bias. The NOS uses a star system to assess the quality of a study, based on selection and comparability of the groups, ascertainment of exposure, and assessment of outcome; the quality of the study was rated as *low* (0–3 stars), *medium* (4–6 starts), or *high* (7–9 stars). Two authors (GHP and SK) evaluated the quality of the selected studies, and any disagreements were resolved through a discussion with the review team. 

### 2.4. Data Analysis

The primary outcomes were hypoprothrombinemia, PT prolongation, and bleeding associated with NMTT-cephalosporin use. Patients with hypoprothrombinemia had elevated PT in their laboratory tests or clinically presenting bleeding [10]. We used the ORs reported in each study. If not reported, we calculated the ORs using the reported rates of hypoprothrombinemia, PT prolongation, and bleeding for each NMTT-cephalosporin. When each study reported data from multiple independent subgroups, we treated each subgroup as a separate study, following the suggested analytic approaches in the literature [11].

The meta-analysis was performed using the Comprehensive Meta-analysis, version 2 (Biostat, Englewood, NJ, USA), and the pooled estimates were presented as ORs with 95% confidence intervals (CIs) derived from the analytical models evaluating the association between NMTT-cephalosporins and study outcomes of hypoprothrombinemia, PT prolongation, and bleeding. Heterogeneity within the included studies was assessed using the *I*^2^ statistic, and we applied either the fixed effects model or the random-effects model, depending on the significance of heterogeneity (P < 0.10 and *I*^2^ ≥ 50%).

## 3. Results

### 3.1. Literature Search

**Figure 1:**
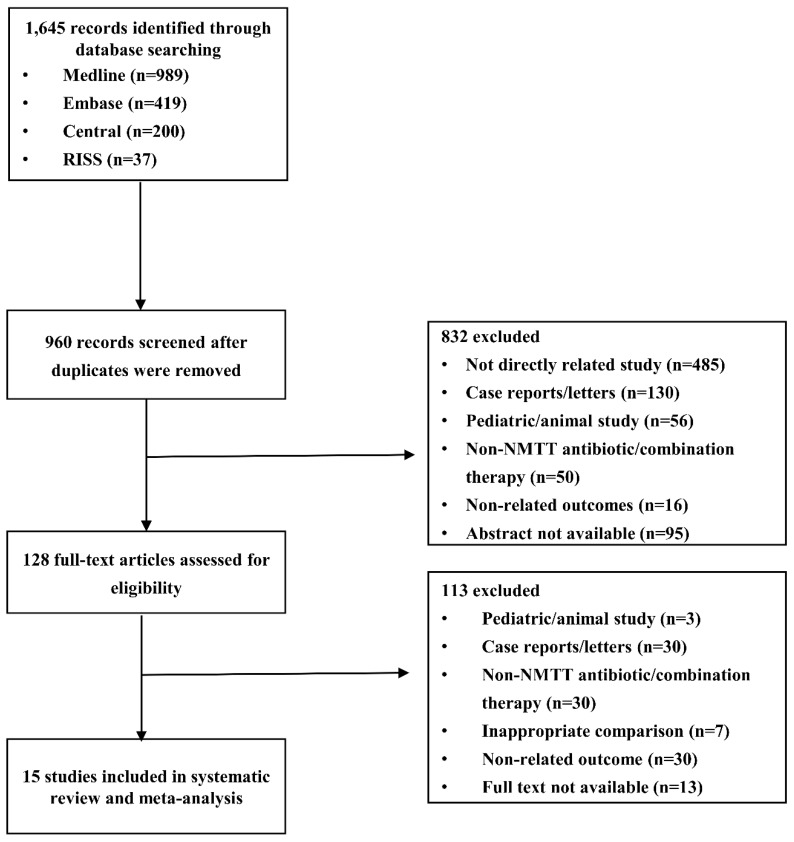
Flow chart of the study selection process.

A total of 1645 articles were identified through a database search. After removing duplicates, 960 records remained, and of those, 128 articles were selected for full-text review. After a full-text review, 113 articles were excluded due to the reasons summarized in Figure 1. The remaining 15 studies were included in the final meta-analysis. They were five RCTs [12,13,14,15,16], four prospective cohort studies [17,18,19,20], five retrospective cohort studies [21,22,23,24,25], and one case-control study [26], published between 1984 and 2016. A total of five NMTT-cephalosporins were included in the analysis; cefamandole [13,23,24], cefoperazone [20,22,25,26], cefotetan [15,16,18,19,20], cefmetazole [26], and moxalactam [12,14,17,21,22,24]. 

### 3.2. Study Characteristics and Quality

The majority of studies (n = 9) [12,13,16,19,20,21,22,23,24] included in our review were conducted in the US, with the exception of four [14,17,18,25] that were conducted in England, one in South African [15], and one in Taiwan [26]. The studies reported that cephalosporins were prescribed for the following indications: pneumonia, preoperative and postoperative antibiotic regimen, and sepsis. Among the 15 studies, moxalactam (latamoxef) was the most commonly used NMTT-cephalosporin (6 studies), followed by cefotetan (5 studies). A total of 13 [12,13,14,15,16,18,19,20,21,22,23,24,25] and 11 studies [12,14,16,17,19,20,21,22,24,25,26] out of 15 studies reported PT prolongation and bleeding events, respectively. As the control group, most of the studies included patients who received other types of antibiotics, including non-NMTT-cephalosporins, aminoglycosides, and penicillin. The characteristics of these 15 studies included in the meta-analysis are summarized in Table 1.

With respect to quality assessment, four studies [23,24,25,26] were considered as high quality, and the remaining six studies [17,18,19,20,21,22] were considered as medium quality, due to insufficient descriptions on the study population and methods for controlling the confounding factors, such as age, sex, clinical severity of infection, and bleeding risks. Detailed information regarding quality assessment is described in Tables S4 and S5. 

**Figure 2:**
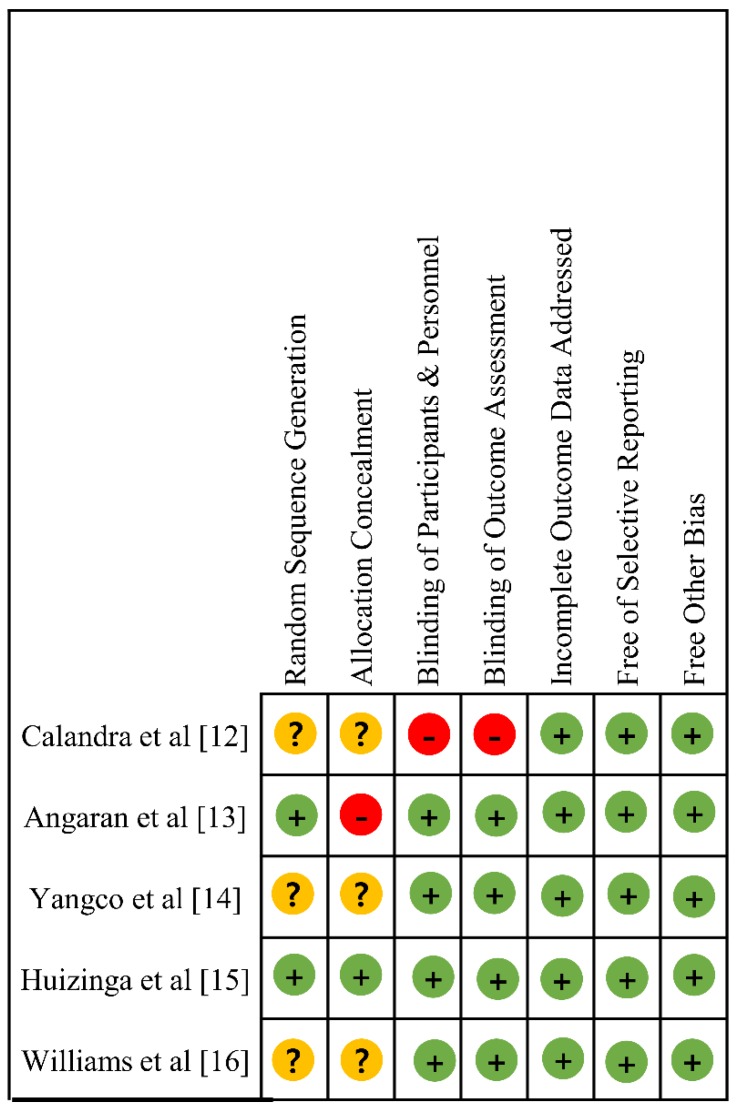
Risk of bias in the included randomized controlled studies.

The risk of bias of five RCTs [12,13,14,15,16] using the RoB algorithm is displayed in Figure 2. All five studies showed a low probability of bias in three domains: (1) incomplete outcome data addressed, (2) free of selective reporting, and (3) free of other bias. Other than the study by Calandra et al. [12], which adopted a non-blinded design, the outcomes from the remaining four studies [13,14,15,16] were not likely to be influenced by blinding. However, the qualities of most studies were “low” in random sequence generation [12,14,16] and allocation concealment [12,13,14,16].

### 3.3. Meta-Analysis

**Figure 3:**
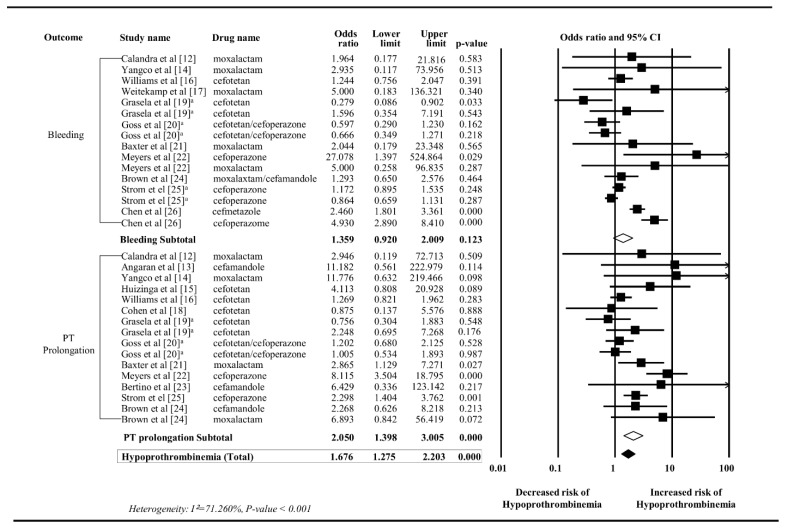
Forest plots of odds ratio for bleeding, PT prolongation, and hypoprothrombinemia associated with NMTT-cephalosporin compared with non-NMTT-cephalosporin. NMTT, N-methylthiotetrazole side chain. ^a^ Multiple control groups from the study were treated independently in the meta-analysis.

The findings of the meta-analysis are summarized in Figure 3. Eleven studies provided 16 data points suitable for a meta-analysis with bleeding outcomes. No significant association was found between NMTT-cephalosporins and bleeding (OR 1.359, 95% CI 0.920–2.009). A random-effects model was applied due to the statistical significance of heterogeneity across the studies (*I^2^* = 78.73%, *P* < 0.001). 

A total of 16 data points from 13 studies were analyzed to evaluate the association between NMTT-cephalosporins and PT prolongation. The use of NMTT-cephalosporins significantly increased PT (OR 2.050, 95% CI 1.398–3.005), with high heterogeneity (*I^2^* = 53.903%, *P* = 0.005). Subgroup analyses revealed that only cefoperazone (OR 4.117, 95% CI 1.200–14.126), cefamandole (OR 3.247, 95% CI 1.083–9.733), and moxalactam (OR 3.606, 95% CI 1.633–7.963) were statistically significant in PT prolongation (Figure S6). 

**Figure 4:**
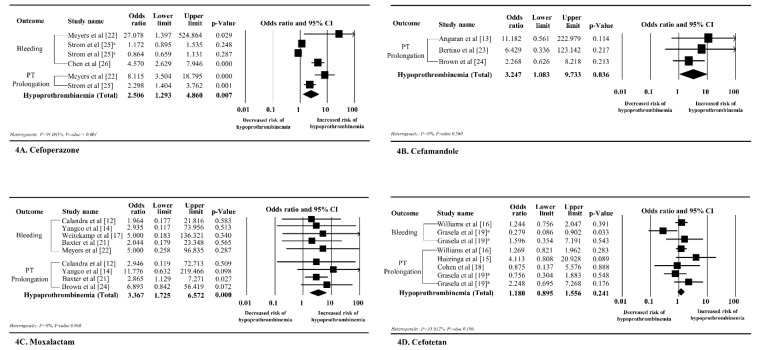
Subgroup analyses of hypoprothrombinemia and each NMTT-cephalosporins. NMTT, N-methylthiotetrazole side chain. ^a^ Multiple control groups from the study were treated independently in the meta-analysis.

A total of 32 events of hypoprothrombinemia, combining bleeding and PT prolongation, from 15 studies were included for the analysis. There were statistically significant associations between NMTT-cephalosporins and hypoprothrombinemia (OR 1.676, 95% CI 1.275–2.203). When further subgroup analyses were conducted by each NMTT-cephalosporin, cefoperazone (OR 2.506, 95% CI 1.293–4.860), cefamandole (OR 3.247, 95% CI 1.083–9.733), and moxalactam (OR 3.367, 95% CI 1.725–6.572) were significantly associated with hypoprothrombinemia than non-NMTT-cephalosporins, but the risk was not significant with cefotetan (OR 1.180, 95% CI 0.895–1.556) (Figure 4). A few studies [20,24] were excluded from the subgroup analysis, because there was more than one NMTT-cephalosporin included in the study, and the number of patients in each treatment group was not clear. Cefmetazole was also excluded from the subgroup analysis because there was only one study [26]. 

## 4. Discussion

To the best of our knowledge, this is the first systematic review and meta-analysis evaluating the associations between NMTT-cephalosporins and hypoprothrombinemia, PT prolongation, and bleeding. The findings in this study suggest that there may be a significantly increased risk of hypoprothrombinemia and PT prolongation from using NMTT-cephalosporins. Of the five NMTT-cephalosporins, cefoperazone, cefamandole, and moxalactam appear to be associated with a higher incidence of hypoprothrombinemia and PT prolongation. However, our study did not find a statistically significant relationship between NMTT-cephalosporins and bleeding events. 

Hypoprothrombinemia, a disease characterized by a deficiency of the clotting factor prothrombin, presents an elevated PT level and a prolonged activated partial thromboplastin time (aPTT) [10]. Bleeding is a common clinical manifestation of hypoprothrombinemia [10], but numerous other factors, such as the patient’s underlying characteristics and coexisting medical conditions, may also contribute to bleeding tendencies. As the information on the exact frequency of bleeding symptoms in patients with hypoprothrombinemia is lacking, patients who have abnormal lab test results, with or without symptoms, should require further investigation for underlying iatrogenic causes. Moreover, coagulation abnormalities commonly occur in hospitalized patients who are being treated with antibiotics [27], and bleeding from wound sites was observed in postsurgical patients when NMTT-cephalosporin was administered as surgical prophylaxis [17]. Thus, proper guidelines on the initial evaluation of bleeding symptoms, monitoring plans for inpatients and postsurgical patients with antibiotics, especially NMTT-cephalosporins, are needed.

One of the strengths of our study was the inclusion of a variety of NMTT-cephalosporins to quantify the risk of each NMTT-cephalosporin on the specific outcomes. Although the exact mechanisms of NMTT-cephalosporins inducing the risk of hypoprothrombinemia, remain mostly undefined, two potential mechanisms involving the depletion of vitamin K-dependent clotting factors by the NMTT-side chain have been suggested in previous literature: 1) inhibition of vitamin K-dependent gamma-carboxylation of glutamic acid [5]; 2) eradication of vitamin K-producing intestinal microflora [7]. However, like cefotetan in our subgroup analysis, not all NMTT-cephalosporins have exhibited hypoprothrombinemia, indicating the possibility of additional mechanisms or influencing factors of NMTT-cephalosporins inducing hypoprothrombinemia. Based on the in vitro and in vivo study comparing the pharmacokinetic profiles of moxalactam, cefoperazone, and cefotetan [28], the concentrations of NMTT cleaved from cefotetan showed the lowest as compared to those from other parent cephalosporins. These findings suggest varying potentials in inducing hypoprothrombinemia within NMTT-cephalosporins. Thus, more confirmatory studies are needed to investigate the detailed mechanisms, NMTT concentration-response relationship, or at least the biological factors responsible for NMTT-cephalosporin-associated hypoprothrombinemia.

To obtain reliable results, we limited the study design to the top three highest levels of evidence: RCT, cohort studies, and case-control studies [29]. RCT studies have a higher degree of validity, allowing a more reliable evaluation of the drug safety profile. By including observational studies in addition to RCTs, our study attempted to reflect real-world evidence. Moreover, we conducted subgroup analyses to investigate which specific NMTT-cephalosporin influenced PT prolongation and hypoprothrombinemia, i.e., two significant outcomes in our study. Since the risk of ADRs is dependent on the individuals’ clinical background, dietary intake, such as high vitamin K-containing foods, and/or concurrent medications that are known to induce bleeding, these factors should be considered in addition to the use of NMTT-cephalosporins when making clinical recommendations.

Unlike some European countries [30,31,32], the US [3], and Japan [33] with relatively low use of NMTT-cephalosporins, all five NMTT-cephalosporins are approved and actively used in South Korea [2]. For countries like South Korea that are actively using NMTT-cephalosporins, we believe that concerted efforts in improving the awareness about the adverse outcomes and updating the safety issues based on the data from continuous surveillance are important. From that perspective, the Antimicrobial Stewardship Program (ASP) operated by the hospital’s multidisciplinary team could play a critical role in educating and monitoring potential safety concerns with respect to NMTT-cephalosporins in those countries.

Our study has a few limitations. First, the dose-response relationship was not fully addressed because we assessed a single cephalosporin regimen. Jones et al. [34] compared two dosing regimens of cefoperazone plus mezlocillin and found that hypoprothrombinemia was more frequent in a higher dose regimen. Second, heterogeneity of the comparison groups and various definitions of the outcomes, such as PT prolongation and bleeding, were additional limitations of our study. The definitions of PT prolongation were different between studies varying from PT increases over 2 seconds from the baseline, 5 seconds over baseline, or final PT measurement over 14 seconds to a measured PT converted into the International Normalized Ratio. The adverse reaction of bleeding also varied, presenting as clinical signs and symptoms, such as hematemesis, rectal bleeding, gastrointestinal bleeding, or a decrease in hemoglobin level. These might result in either over- or under-estimation. Third, our meta-analysis could only produce unadjusted ORs because each study controlled different confounding variables and additional risk factors that may have affected PT levels or bleeding, such as underlying diseases, concurrent medication use, procedures, or nutritional status of patients, could not be controlled in the analysis. Lastly, the included studies except for Chen et al. [26] were conducted before 2000. While the practice guidelines for the use of antibiotics, prescribing patterns, patient population, and other factors related to the health care environment have changed in the last decades, findings from our study could not reflect these factors. In addition, the adverse events associated with the NMTT-cephalosporins related to coagulation have been continuously reported until recently [35,36]. Therefore, our findings should be interpreted while considering these factors, and future studies incorporating new updates are needed.

## 5. Conclusions

We conducted a systematic review and meta-analysis of both experimental and observational studies to evaluate the association between NMTT-cephalosporins and hypoprothrombinemia, PT prolongation, and bleeding in patients. Our study concludes that an increased risk of hypoprothrombinemia and PT prolongation were significantly associated with NMTT-cephalosporin use. However, bleeding was not significantly increased with NMTT-cephalosporins. Healthcare professionals should be aware of the potential risk of NMTT-cephalosporins on hypoprothrombinemia and carefully monitor patients who have additional underlying risk factors for bleeding. To ensure safe drug prescribing, drug safety data should be systematically and continuously updated and reviewed.

## Figures and Tables

**Table 1:** Characteristics of included studies.

Study	Country	Study Design	Population Characteristics	Total Population	Intervention ^a^	Control	Outcomes and Definitions		QA Score ^c^
							PT ^b^	Bleeding	
Calandra et al. (1984) [12]	United States	RCT	Infections	441	Moxalactam 2g q8h for 5–14 days	Imipenem/cilastatin	PT increases by 3s or 25%	Hemorrhagic colitis, hemoperitoneum, GI hemorrhage	–
Angaran et al. (1987) [13]	United States	RCT	Patients who were scheduled to have cardiac valve replacement surgery	40	Cefamandole 2g q6h	Vancomycin	PT_3_^d^ ≥ 32s	─	–
Yangco et al. (1987) [14]	England	RCT	Patients with suspected bacterial pneumonia	92	Latamoxef 2–4g q12h for mild renal impairment, 1-2g q12-24h for moderate to severe renal impairment	Ceftizoxime	Abnormality in clinical laboratory value (PT/PTT)	Adverse effects (hematemesis)	–
Huizinga (1988) [15]	South Africa	RCT	Severe intra-abdominal sepsis	96	Cefotetan 2g q12h	Ampicillin + gentamicin + metronidazole	PT > 5s	─	–
Williams et al. (1991) [16]	United States	RCT	Infections	1109	Cefotetan	Non-NMTT ABx ^e^	PT > 5s above upper limit of normal range	Clinical bleeding episodes	–
Weitekamp et al. (1985) [17]	England	Prospective cohort	Healthy volunteers	10	Latamoxef 4g q24h, 2g q8h, 4g q8h	Cefotaxime	─	Clinical bleeding	5
Cohen et al. (1988) [18]	England	Prospective cohort	Patients with intra-abdominal sepsis who underwent surgery	20	Cefotetan	Cephadrin+ metronidazole or gentamicin + penicillin + metronidazole	INR^f^ > 1.2	─	4
Grasela et al. (1989) [19]	United States	Prospective cohort	Patients who require IV antibiotic therapy for intra-abdominal or obstetric-gynecologic process	970	Cefotetan	1) Aminoglycoside + antianaerobic 2) cefoxitin	PT > 2s over baseline	Requiring transfusion	6
Goss et al. (1992) [20]	United States	Prospective cohort	Patients with cancer, fever, granulocytopenia, intraabdominal infection, nosocomial pneumonia	460	Cefotetan or cefoperazone	Non-NMTT ABx ^g^	PT > 14s	Decrease in hemoglobin 10g/L or greater over a 24-hour period/required vitamin K/transfusion	6
Baxter et al. (1985) [21]	United States	Retrospective cohort	Abdominal sepsis	94	Moxalactam 2g q8h	Tobramycin + clindamycin	PT > 2s over baseline	Requiring transfusion of 1 to 2 units of packed red cells	5
Meyers et al. (1985) [22]	United States	Retrospective cohort	Infections	4948	Moxalactam or cefoperazone	Ceftazidime	Increased PT	Clinical bleeding	4
Bertino et al. (1986) [23]	United States	Retrospective cohort	─ ^h^	41	Cefamandole	Nafcillin/oxacillin	PT > 2s above the highest control end point	─	7
Brown et al. (1986) [24]	United States	Retrospective cohort	─	755 for bleeding 1318 for increased PT	Cefamandole or moxalactam	Penicillin + cefoxitin	Increase in PT/PTT	Presence of grossly observable blood	7
Strom et al. (1999) [25]	England	Retrospective cohort	Infections	853	Cefoperazone	1) Ceftizoxime/ Cefotaxime 2) Ceftazidime	PT + 2s, 5s, 15s PT×1.25, 1.5, 2 from baseline	Microscopically observed blood, grossly observed blood without transfusions, grossly observed blood with transfusions, cerebral hemorrhage, death from bleeding	7
Chen et al. (2016) [26]	Taiwan	Case-control	Patients hospitalized due to a hemorrhagic event subsequent to the use of antibiotics in ER	6191	Cefoperazone or cefmetazole	Non-NMTT ABx ^i^	─	Diagnosis code of hemorrhage	8

^a^ If study provided dosing for their intervention or control. ^b^ PT is measured in seconds. ^c^ The quality of the observational studies and randomized controlled trials was assessed using a modified Newcastle–Ottawa Scale (NOS) and Risk of Bias (RoB) tool, respectively. The NOS score is presented in the table by the number of stars, and the results of RoB assessment are presented separately in Figure 2. ^d^ PT_3_ is measured at 16 hours after a second warfarin dose. ^e^ Non-NMTT antibiotics include cefotaxime, ampicillin/metronidazole plus gentamicin, cefoxitin plus metronidazole, cefoxitime, and cephradine plus metronidazole. ^f^ INR is calculated from PT results [(PT_test_/PT_normal_)^International Sensitivity Index^]. ^g^ Non-NMTT antibiotics include 1) cefoxitin, ceftriaxone, ceftizoxime, ceftazidime, cefotaxime; 2) aminoglycoside plus anti-anaerobic, and aminoglycoside plus penicillin. ^h^ Study did not provide clinical indications of treated patients. ^i^ Non-NMTT antibiotics include amoxicillin/clavulanic acid, ampicillin-sulbactam, cefuroxime, ceftriaxone, and cefotaxime. Abbreviation: ABx, antibiotics; IV, intravenous; NMTT, N-methylthiotetrazole side chain; PT, prothrombin time; PTT, partial thromboplastin time; INR, International Normalized Ratio; QA, quality assessment; S, seconds; ER, emergency room.

## Supplementary Materials

The following are available online at https://www.mdpi.com/1660-4601/16/20/3937/s1; Table S1: Search strategy, Table S2: National Evidence-based healthcare Collaborating Agency (NECA) RoB guidelines, Table S3: Criteria for modified Newcastle–Ottawa Scale, Table S4: Newcastle–Ottawa Scale (NOS) for assessing the quality of cohort studies, Table S5: Newcastle–Ottawa Scale (NOS) for assessing the quality of case-control, case-population and case/noncase studies. Figure S6: Subgroup analyses of PT prolongation and NMTT-cephalosporins.
