# Supplementary material for: The Association Between Cephalosporin and Hypoprothrombinemia: A Systematic Review and Meta-Analysis
Source: Int J Environ Res Public Health. 2019 Oct 16;16(20):3937. doi: 10.3390/ijerph16203937 (PMC6843226; doi:10.3390/ijerph16203937)
Supplement: Supplementary file 1 [file ijerph-16-03937-s001.pdf]

Table S1. Search strategy.

<table>
<tr><th colspan="2">PubMed</th></tr>
<tr><td>#1</td><td>"Cephalosporins"[Mesh] OR "cephalosporin"[All Fields] OR “cephalosporins”[All Fields]</td></tr>
<tr><td>#2</td><td>cefamandole OR cefmenoxime OR cefmetazole OR cefoperazone OR cefotetan OR moxalactam</td></tr>
<tr><td>#3</td><td>"Hypoprothrombinemias"[Mesh] OR "hypoprothrombinemia"[All Fields] OR "hypoprothrombinemias"[All Fields] OR "hypoprothrombinaemia"[All Fields] OR “hypoprothombinaemias”[All Fields]</td></tr>
<tr><td>#4</td><td>"Hemorrhage/chemically induced"[Mesh] OR "bleeding"[All Fields] OR "hemorrhage"[All Fields] OR "hemorrhages"[All Fields] OR "haemorrhage"[All Fields]</td></tr>
<tr><td>#5</td><td>#1 AND #3</td></tr>
<tr><td>#6</td><td>#1 AND #4</td></tr>
<tr><td>#7</td><td>#2 AND #3</td></tr>
<tr><td>#8</td><td>#2 AND #4</td></tr>
<tr><th colspan="2">EMBASE</th></tr>
<tr><td>#1</td><td>ʻcephalosporin’/exp OR cephalosporin OR cephalosporins</td></tr>
<tr><td>#2</td><td>'cefamandole'/exp OR cefamandole OR 'cefmenoxime'/exp OR cefmenoxime OR 'cefmetazole'/exp OR cefmetazole OR 'cefotetan'/exp OR cefotetan OR 'latamoxef'/exp OR latamoxef OR moxalactam OR 'cefoperazone'/exp OR cefoperazone</td></tr>
<tr><td>#3</td><td>'hypoprothrombinemia'/exp OR hypoprothrombinemia OR hypoprothrombinemias OR hypoprothrombinaemia OR hypoprothrombinaemias</td></tr>
<tr><td>#4</td><td>'bleeding'/mj (49985)</td></tr>
<tr><td>#5</td><td>#1 AND #3</td></tr>
<tr><td>#6</td><td>#1 AND #4</td></tr>
<tr><td>#7</td><td>#2 AND #3</td></tr>
<tr><td>#8</td><td>#2 AND #4</td></tr>
<tr><th colspan="2">Cochrane</th></tr>
<tr><td>#1</td><td>[cephalosporins/explode all trees] OR cephalosporins OR cephalosporin</td></tr>
<tr><td>#2</td><td>[cefamandole/explode all trees] OR [cefmenoxime/explode all trees] OR [cefmetazole/explode all trees] OR [cefoperazone/explode all trees] OR [cefotetan/explode all trees] OR [moxalactam/explode all trees] OR cefamandole OR cefmenoxime OR cefmetazole OR cefoperazone OR cefotetan OR moxalactam</td></tr>
<tr><td>#3</td><td>[hypoprothrombinemias/explode all trees] OR hypoprothrombinemias OR hypoprothrombinemia OR hypoprothrombinaemia OR hypoprothrombinaemias</td></tr>
<tr><td>#4</td><td>[hemorrhage/explode all trees] OR hemorrhage OR hemorrhages OR haemorrhages OR haemorrhage OR bleeding</td></tr>
<tr><td>#5</td><td>#1 AND #3</td></tr>
<tr><td>#6</td><td>#1 AND #4</td></tr>
<tr><td>#7</td><td>#2 AND #3</td></tr>
<tr><td>#8</td><td>#2 AND #4</td></tr>
<tr><th colspan="2">RISS</th></tr>
<tr><td>#1</td><td>cephalosporin | cephalosporins<AND> hypoprothrombinemia | hypoprothrombinemias | hypoprothrombinaemia</td></tr>
<tr><td>#2</td><td>cephalosporin | cephalosporins <AND> bleeding | hemorrhage | hemorrhages | haemorrhage | haemorrhages</td></tr>
<tr><td>#3</td><td>cefamandole | cefmenoxime | cefmetazole | cefoperazone | cefotetan | moxalactam <AND> hypoprothrombinemia | hypoprothrombinemias | hypoprothrombinaemia</td></tr>
<tr><td>#4</td><td>cefamandole | cefmenoxime | cefmetazole | cefoperazone | cefotetan | moxalactam <AND> bleeding | hemorrhage | hemorrhages | haemorrhage | haemorrhages</td></tr>
</table>

Table S2. National Evidence-based healthcare Collaborating Agency (NECA) RoB guidelines.

Item	Criteria	Risk
**SELECTION BIAS**		
1. Random sequence generation	Sequence generation process such as:<br>- Random number table<br>- Computer random number generator<br>- Coin tossing<br>- Shuffling cards or envelopes	Low
	Non-random component in the sequence generation process such as:<br>- Odd or even date of birth<br>- Date of admission<br>- Hospital record number<br>- Results of laboratory test<br>- Allocation by clinician/participants	High
	Insufficient information	Unclear
2. Allocation concealment	Participants and investigators enrolling participants could not foresee assignment because one of the following, or an equivalent method, was used to conceal allocation	Low
	Participants or investigators enrolling participants could possibly foresee assignments and thus introduce selection bias	High
	Insufficient information	Unclear
**PERFORMANCE BIAS**		
3. Blinding of participants and personnel	Blinding of participants and key study personnel ensured or review author judge that the outcome is not likely influenced by lack of blinding	Low
	No blinding or incomplete blinding	High
	Insufficient information	Unclear
**DETECTION BIAS**		
4. Blinding of outcome assessment	Blinding of outcome assessment ensured or review author judge that the outcome is not likely influenced by lack of blinding	Low
	No blinding or incomplete blinding	High
	Insufficient information	Unclear
**ATTRITION BIAS**		
5. Incomplete outcome data	- No missing outcome data<br>- Reasons for missing outcome data unlikely to be related to true outcome<br>- Missing outcome data balanced in numbers across intervention groups, with similar reasons for missing data across groups;	Low
	Reason for missing outcome data likely to be related to true outcome, with either imbalance in numbers or reasons for missing data across intervention groups	High
	Insufficient information	Unclear
**REPORTING BIAS**		

6. Selective reporting	All of the study's pre-specified outcomes that are of interest has been reported	Low
	Not all of the study's pre-specified outcomes that are of interest has been reported	High
	Insufficient information	Unclear
**OTHER**		
7. Other bias	The study appears to be free of other bias	Low
	There is at least one important risk of bias	High
	Insufficient information	Unclear

Table S3. Criteria for modified Newcastle-Ottawa Scale.

Item	Criteria	Score
**SELECTION**		
1. Representativeness of the exposed cohort	Population-based study, random recruitment of participants, or consecutive enrollment of participants	One star
	Selected group of users	Zero star
	No description	Zero star
2. Selection of non-exposed cohort	Drawn from same source as exposed cohort	One star
	Drawn from different source	Zero star
	No description	Zero star
3. Ascertainment of exposure	Medical records or structured interview	One star
	Self-report	Zero star
	No description	Zero star
4. Demonstration that outcome of interest was not present at start of study	Yes	One star
	No	Zero star
**COMPARABILITY**		
1. Comparability of cohorts on the basis of the design or analysis	Adjustment or exclusion of the confounding factors for bleeding	One star
	Adjustment or control of the confounding factors for patient characteristics	One star
	No description	Zero star
**OUTCOME**		
1. Ascertainment of outcome	Standardized assessment or confirmation of bleeding or PT prolongation in the medical record	One star
	Self-report	Zero star
	No description	Zero star
2. Enough period of follow-up for outcome of interest to occur	Yes	One star
	No	Zero star
3. Adequacy of follow-up of cohorts	Complete follow up of more than 90% of enrolled participants	One star
	Follow up rate less than 90% and no description of those lost	Zero star
	No description	Zero star

Table S4. Newcastle-Ottawa Scale (NOS) for assessing the quality of cohort studies.

Study	Selection				Comparability	Outcomes		
	Representativeness of exposed cohort	Selection of the nonexposed cohort	Ascertainment of exposure	Demonstration that outcome of interest was not represent at the start of the study	Comparability of Cohort	Assessment of outcome	Was follow-up long enough for outcomes to occur	Adequacy of follow up of cohorts
Weitekamp et al. 1985 [17]	–	–	★	★	–	★	★	★
Cohen et al. 1988 [18]	–	–	★	★	–	★	★	–
Grasela et al. 1989 [19]	–	–	★	★	★	★	★	★
Goss et al. 1992 [20]	–	–	★	★	★★	★	★	–
Baxter et al. 1985 [21]	★	–	★	–	–	★	★	★
Meyers et al. 1985 [22]	–	–	★	–	–	★	★	★
Bertino et al. 1986 [23]	★	★	★	★	–	★	★	★
Brown et al. 1986 [24]	★	★	★	★	–	★	★	★
Strom et al. 1999 [25]	★	★	★	–	★	★	★	★

Table S5. Newcastle-Ottawa Scale (NOS) for assessing the quality of case-control, case-population and case/noncase studies.

	Selection				Comparability	Exposure		
Study	Is the case definition adequate	Representativeness of cases	Selection of controls	Definition of controls	Study controls for important factor or additional factor	Ascertainment of exposure	Same method of ascertainment cases and controls	Nonresponse rate
Chen et al. 2006 [26]	★	★	–	★	★★	★	★	★

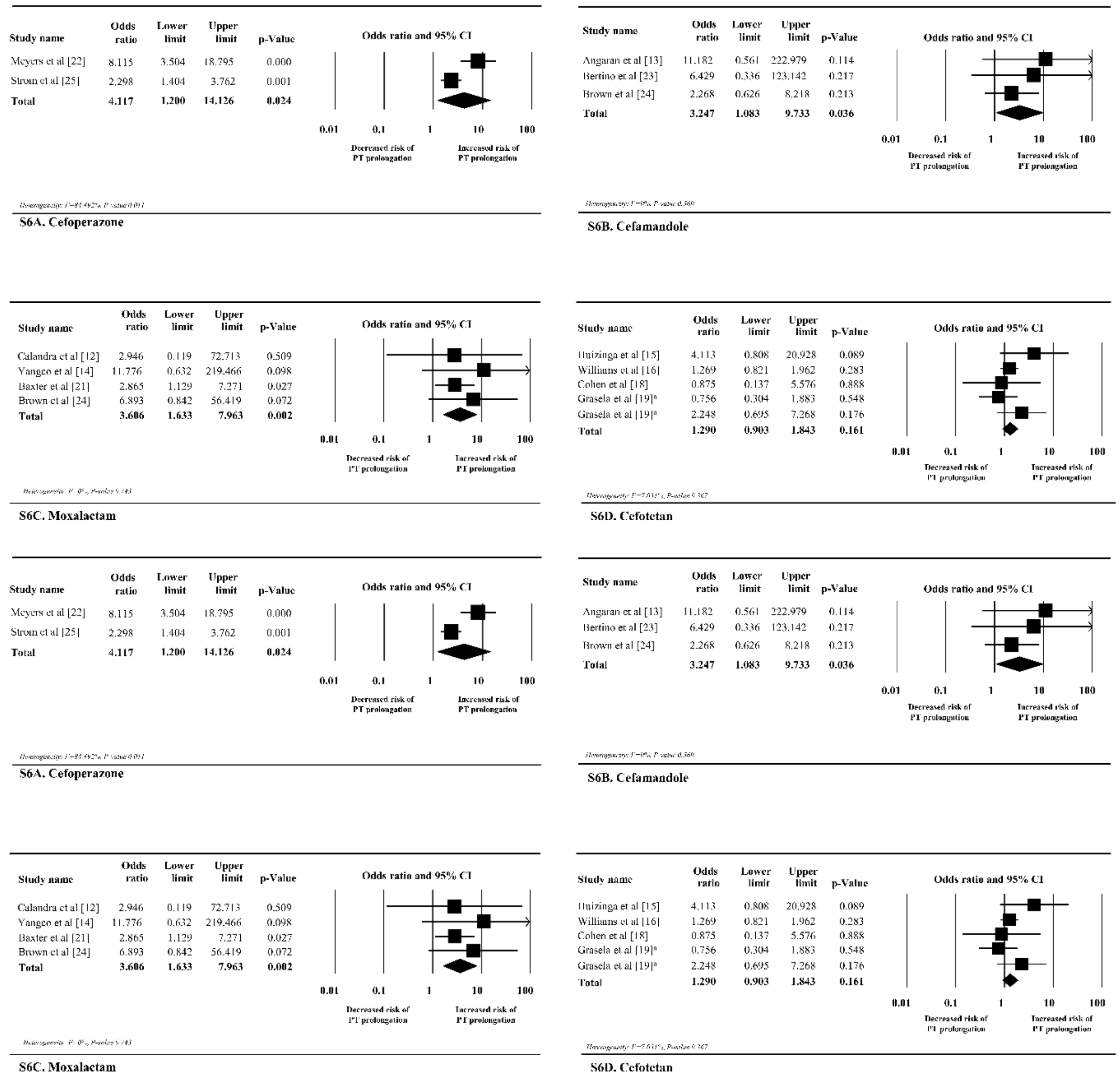

Figure S6. Subgroup analyses of PT prolongation and NMTT-cephalosporins.

NMTT, N-methylthiotetrazole side chain.

[a] Multiple control groups from the study were treated independently in the meta-analysis.
